# CD138-Positive Plasma Cells in the Endometrium of Women with a Caesarean Scar Defect

**DOI:** 10.3390/diagnostics16213472

**Published:** 2026-10-24

**Authors:** Katharina Rathberger, Maurice Kappelmeyer, Johannes Jungbauer-Oberheinrich, Maximilian Rauh, Mehmet Vural, Emilia Solano, Angela Köninger, Tilman Born

**Affiliations:** 1 Department of Obstetrics and Gynecology, University Hospital St. Hedwig of the Order of St. John, University of Regensburg, Steinmetzstraße 1-3, 93049 Regensburg, Germanytilman.born@klinik.uni-regensburg.de (T.B.); 2 Laboratory of Translational Perinatology, Department of Gynecology and Obstetrics, Medical Faculty, University of Regensburg, 93053 Regensburg, Germany

**Keywords:** caesarean scar defect, caesarean scar disorder, chronic endometritis, CD138, plasma cells, infertility

## Abstract

**Objective**: This study seeks to assess the prevalence of CD138-positive plasma cells in endometrial biopsies from women undergoing surgery for symptomatic caesarean scar defect (CSD) and examine associations with niche morphology, defect size, symptoms, and surgical approach. **Study design**: In this prospective single-centre study, women undergoing surgery for symptomatic CSD between January 2024 and June 2026 were included if an endometrial biopsy had been assessed for CD138. Plasma-cell counts were categorised as negative (0 cells/HPF), borderline (1–4 cells/HPF), or elevated (≥5 cells/HPF). Preoperative niche morphology was assessed by transvaginal ultrasound. Associations with CD138 status were tested using Mann–Whitney U and Fisher’s exact tests and multivariable logistic regression. **Results**: Of 235 surgically treated women, 196 had an assessable CD138 result. CD138 positivity was present in 26 (13.3%); 19 (9.7%) had 1–4 cells and seven (3.6%) > 4 cells. CD138 status was not associated with defect size, uterine position, inclusion cysts, concomitant peritoneal endometriosis, surgical approach, or any recorded symptom. The absence of associations persisted after multivariable adjustment. **Conclusions**: Endometrial CD138-positive plasma cells were present in a minority of women with symptomatic CSD and did not reflect defect morphology, severity, or symptom burden. Routine CD138 testing cannot be justified on the basis of niche subtype or defect size.

## 1. Introduction

Caesarean scar defects (CSDs), also termed uterine niches or isthmoceles, are common long-term sequelae of caesarean delivery. With global caesarean section (CS) rates continuing to rise, their clinical relevance is increasing [1]. A niche is sonographically defined as an indentation of at least 2 mm at the site of the hysterotomy scar and is detected in approximately 60% of women after caesarean delivery, depending on the imaging method used [2,3]. When a sonographically confirmed niche is accompanied by characteristic symptoms, including postmenstrual spotting, pain, abnormal discharge, or infertility, the condition is termed caesarean scar disorder (CSDi) [4,5].

The pathophysiology of CSDi is likely multifactorial. In addition to impaired myometrial healing and menstrual blood retention, local epithelial loss, fragile vessels, altered uterine contractility, microbial colonization, and chronic inflammation have been proposed as mechanisms linking the defect to abnormal bleeding and impaired fertility [6,7,8,9,10,11,12]. CD138 immunohistochemistry improves the detection of endometrial plasma cells compared with conventional histology, but there is no universally accepted threshold for chronic endometritis. Published definitions range from any detectable plasma cell to at least five CD138-positive cells per HPF, and low-level findings may have uncertain clinical relevance [13,14,15,16].

Evidence specifically relating CSD to chronic endometritis remains inconsistent. Some studies reported more frequent or more pronounced plasma-cell infiltration in scar tissue or in women with a niche [9,10,11,12,17], whereas other data did not show an association between caesarean delivery and chronic endometritis in the endometrial cavity [10,18]. Importantly, tissue obtained directly from the scar and biopsy specimens from the uterine cavity represent different anatomical compartments and should not be interpreted interchangeably.

Related studies have linked CSDi to extragenital endometriosis, altered uterine microbial profiles, bacterial colonization of the defect, and microbiome changes after hysteroscopic treatment [19,20,21,22]. These observations support an inflammatory component but do not establish whether inflammation at the scar is accompanied by diffuse plasma-cell infiltration in the uterine cavity.

We assessed the prevalence of CD138-positive plasma cells in endometrial biopsies from women undergoing surgery for symptomatic CSD and examined associations with CSD morphology, defect size and resulting surgical approach, clinical symptoms, and concomitant findings. The clinical aim was to determine whether CSD morphology or size could justify routine CD138 analysis in CSDi.

## 2. Materials and Methods

This prospective, single-centre analysis was conducted at the Department of Obstetrics and Gynecology, University Hospital St. Hedwig of the Order of St. John, Regensburg, Germany.

Patients were eligible if they underwent surgery for symptomatic CSD between January 2024 and June 2026, had at least one previous CS, were aged ≥18 years and premenopausal, and had a sonographically confirmed niche according to Jordans et al. [3]. Demographic data and CSD symptoms according to the Delphi definition [4] were recorded. Patients were generally examined in the follicular phase using transvaginal contrast sonography (EXEM Foam^®^ IQ Medical Ventures BV, Rotterdam, The Netherlands); contrast was omitted only for very large, completely fluid-filled defects. All preoperative sonographic assessments were performed by a single experienced examiner using a standardized protocol based on the modified Delphi criteria [3]. Because all examinations were performed by one examiner, inter-observer reproducibility was not assessed.

Concomitant findings, including inclusion cysts and uterine position (ante-/retroflexion, torqued uterus), were documented. CD138 plasma-cell analysis was performed in consecutive patients during the study period independently of individual symptoms. Patients with previous plasma-cell analysis for individual reasons and corresponding presurgical treatment and patients without an assessable CD138 result were excluded. The study was approved by the Ethics Committee of the University of Regensburg on 23 June 2021 (21-2427-101), and all participants provided informed consent to participate. No formal sample-size calculation was performed; the study sample comprised all consecutive eligible patients with an assessable CD138 result during the predefined study period. Accordingly, the study was not designed to exclude modest effect sizes, and null findings were interpreted together with their confidence intervals. For the descriptive interval from the most recent caesarean delivery to surgery, the most recent documented caesarean year was used and the interval was calculated in calendar years.

Surgical treatment followed one of two approaches according to defect size, depth, residual myometrium, and uterine morphology [23,24]. Extensive defects with residual myometrium <2 mm were treated by complete niche excision via laparoscopy and mini-laparotomy, sometimes combined with hysteroscopy; coexisting peritoneal endometriosis was removed during the same procedure. Smaller defects with residual myometrium ≥2 mm and an anteflexed uterus were treated by operative hysteroscopy alone. If more than one procedure was performed, the defect-excision sample was analysed.

The allocated procedure therefore reflected defect size and concomitant uterine morphology. The two analysis groups were niche excision (“Regensburg Approach”) and hysteroscopy alone.

CD138 was assessed in a Pipelle^®^ (Laboratoire CCD, Paris, France) endometrial biopsy obtained from the uterine cavity during surgery using the same sampling approach in both groups. Sampling was directed to the endometrial cavity and was not specifically targeted to the niche, because the study aimed to assess plasma-cell infiltration of the endometrial cavity rather than scar tissue. Immunohistochemistry was performed on formalin-fixed, paraffin-embedded sections after heat-induced epitope retrieval in citrate buffer (pH 6.0) using monoclonal mouse anti-CD138 (syndecan-1; clone B-A38; Zytomed Systems, Berlin, Germany; 1:200) with tonsil tissue as positive control. CD138-positive plasma cells were counted per HPF at 400×; four HPFs corresponded to 1 mm^2^. Three board-certified pathologists assessed the sections. Hysteroscopic features suggestive of focal endometritis were not assessed using a predefined standardized protocol and were therefore not analysed.

Because no internationally accepted CD138 threshold exists, findings were categorised as negative (0 cells), borderline elevated (1–4 cells), or elevated (>4 cells), reflecting commonly used criteria of any detectable plasma cell versus at least five cells per HPF [10,13,14,15]. For group comparisons, CD138 status was dichotomised as positive versus negative using the inclusive criterion; the stricter definition was retained descriptively.

Uterine position and niche morphology were classified as anteflexion (type 1), retroflexion (type 2), inclusion cysts (type 3), or a torqued configuration (type 4); variable positions were classified as retroflexion. Inclusion cysts may result from entrapped endocervical epithelium, while retroflexed or torqued positions may impair menstrual drainage and promote blood retention and repetitive tissue injury [25].

Statistical analyses were performed using R version 4.4.1. Continuous variables were summarised by mean, standard deviation, median, and interquartile range; categorical variables were summarised by frequencies. Two-group comparisons used the Mann–Whitney U test for continuous variables and Fisher’s exact test for categorical variables; Kruskal–Wallis tests were used for more than two groups. Multivariable logistic regression assessed niche depth or residual myometrium as predictors of CD138 positivity, adjusting for surgical technique, age, body mass index, and parity. Analyses were based on available cases for each comparison and complete cases for the multivariable models; no imputation of missing data was performed. Adjusted odds ratios with 95% confidence intervals were reported. For Tables 2 and 3, effect estimates are additionally presented with 95% confidence intervals. An RPL-related history was defined as ≥2 pregnancy losses after the most recent caesarean section, using the ESHRE threshold of two pregnancy losses [26]. Sensitivity analyses repeated the principal univariable comparisons using ≥5 versus <5 CD138-positive cells/HPF; no multivariable model was fitted because only seven patients met the stricter threshold. All association analyses were exploratory; no adjustment for multiple comparisons was applied, and *p* values should be interpreted descriptively.

## 3. Results

**Figure 1:**
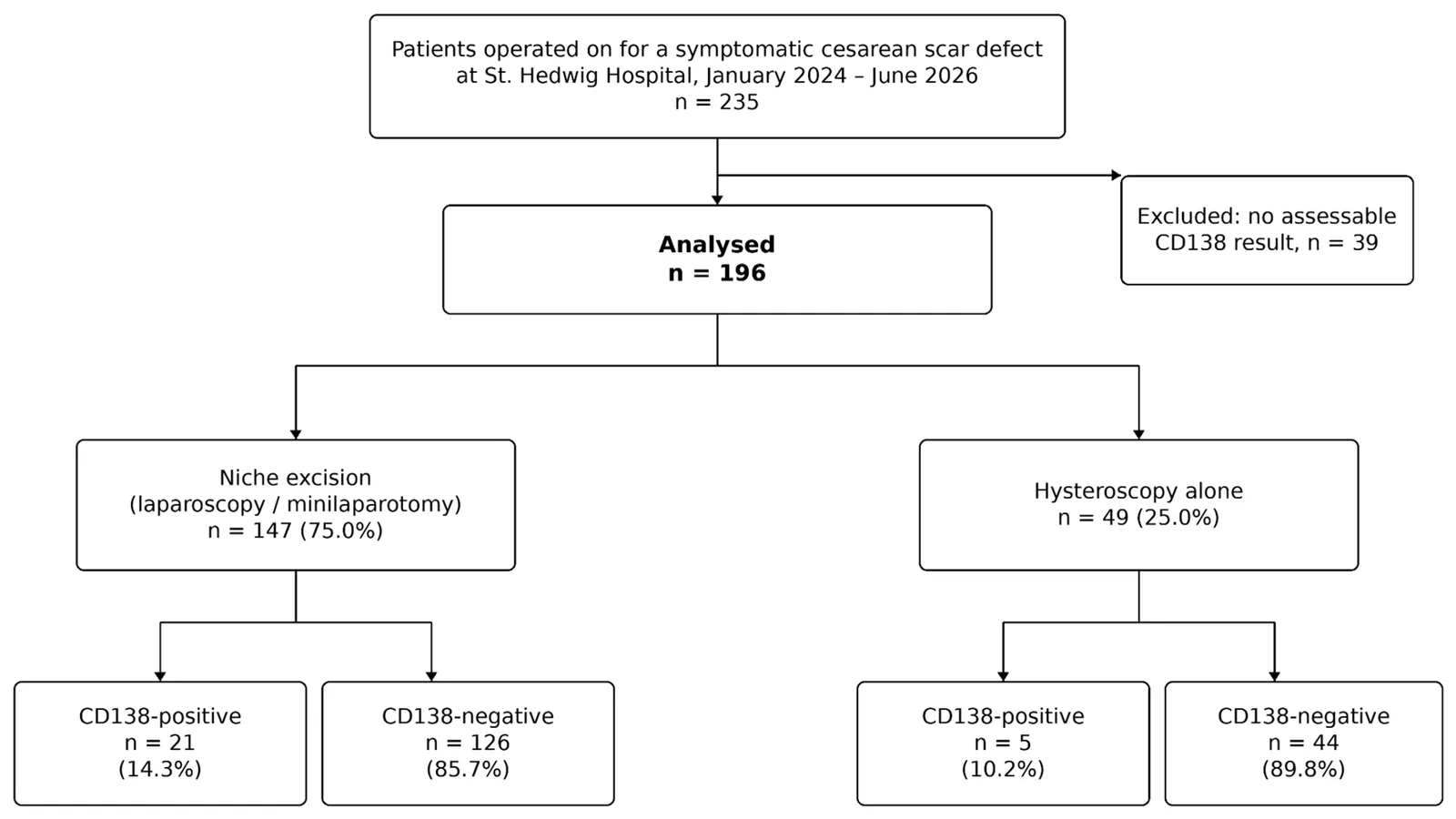
Selection of the study population and distribution of endometrial CD138 findings.

Of 235 surgically treated women, 196 (83.4%) yielded an assessable CD138 result, whereas 39 (16.6%) did not have an assessable study CD138 result and were excluded. Of the 196 included patients, 147 (75.0%) underwent niche excision by laparoscopy/laparotomy, in some cases combined with hysteroscopy (Regensburg Approach), and 49 (25.0%) hysteroscopy alone. The selection of the study population and distribution of CD138 findings are shown in Figure 1. CD138-positive findings were present in 26 patients (13.3%): 19 (9.7%) had 1–4 cells and seven (3.6%) > 4 cells, while 170 (86.7%) were CD138-negative. Among the CD138-positive patients, the median cell count was 3.0 (IQR 2.0–7.8).

Table 1 summarises the cohort characteristics. Most women had undergone one previous CS (152/194; 78.4%), 33 (17.0%) two, and nine (4.6%) three or more; nine (4.6%) had never delivered vaginally. At least one miscarriage was reported by 87/195 women (44.6%), including two or more in 40 (20.5%). Among 176 women with a documented year of the most recent caesarean delivery, the median interval from caesarean delivery to CSD surgery was 4 years (IQR 3–6).

No difference in preoperative morphological niche parameters was observed between CD138-positive and CD138-negative patients (Table 2).

**Table 2:** Preoperative niche morphology according to endometrial CD138 status. Effect estimates are differences in medians with bootstrap 95% confidence intervals; *p* values are from Mann–Whitney U tests.

Parameter (Before Surgery)	CD138-Positive Median (IQR)	CD138-Negative Median (IQR)	n	Effect (95% CI)	*p*
RMT (mm)	0.00 (0.00–2.48)	0.70 (0.00–2.58)	196	−0.70 (−1.25 to 1.55)	0.44
Niche depth (mm)	7.7 (5.1–9.5)	6.9 (4.5–9.1)	192	0.80 (−1.10 to 2.05)	0.65
Proportion of niche in myometrium	1.00 (0.65–1.00)	0.87 (0.69–1.00)	171	0.13 (−0.12 to 0.21)	0.41

Although the two groups differed markedly in defect morphology and size, as intended by the allocation criteria (Table 1), CD138 positivity did not differ significantly between them (14.3% vs. 10.2%; *p* = 0.63, Fisher’s exact test).

None of the recorded symptoms were associated with CD138 status (Table 3). The largest non-significant differences concerned hypermenorrhoea and abnormal vaginal discharge.

**Table 3:** Clinical symptoms according to endometrial CD138 status. Effect estimates are odds ratios with exact 95% confidence intervals; *p* values are from Fisher’s exact test.

Symptom	n	CD138 Positive	CD138 Negative	OR (95% CI)	*p*
Hypermenorrhoea	189	27%	13%	2.48 (0.78–7.14)	0.075
Abnormal discharge	162	24%	40%	0.48 (0.13–1.46)	0.23
Infertility	196	73%	71%	1.13 (0.42–3.39)	>0.9
Dysmenorrhea	195	38%	51%	0.59 (0.23–1.48)	0.29
Dyspareunia	194	35%	38%	0.86 (0.32–2.19)	0.83
Postmenstrual spotting	195	80%	73%	1.48 (0.50–5.35)	0.63

Inclusion cysts were not associated with CD138 status (16.0% in CD138-positive vs. 24.7% in CD138-negative patients; n = 183; *p* = 0.45). Uterine position likewise showed no association, either across the defined categories (n = 195) or for retroflexion versus non-retroflexion (34.6% vs. 32.0%; *p* = 0.82). Concomitant endometriosis was also unrelated to CD138 status (64.0% vs. 57.4%; n = 187; *p* = 0.66).

An RPL-related history was documented in 19/140 patients (13.6%) and at least two previous embryo transfers in 38/87 women with a documented number of transfers (43.7%). CD138 status was associated with neither an RPL-related history (16% vs. 12%; *p* = 0.71) nor ≥2 embryo transfers (11% vs. 20%; *p* = 0.25); only three and four CD138-positive patients, respectively, were present in these subgroups.

In the sensitivity analysis using the stricter threshold of ≥5 cells/HPF versus <5 cells/HPF, no significant associations were identified for niche dimensions, uterine position, inclusion cysts, concomitant endometriosis, surgical approach, reproductive-history variables, or clinical symptoms (Supplementary Table S1).

In multivariable logistic regression, neither niche depth nor residual myometrium independently predicted CD138 positivity after adjustment (Table 4).

## 4. Discussion

In this prospective surgical cohort, endometrial CD138 positivity was infrequent and was not associated with the anatomical or clinical phenotype of CSD, including after multivariable adjustment. These findings suggest that endometrial plasma-cell infiltration does not track anatomical severity and raise the hypothesis that CSDi-associated inflammation may include an injury-driven component not adequately captured by cavity CD138 staining.

The niche may retain menstrual blood beyond menstruation, exposing scar tissue to erythrocytes, cellular debris, and haemoglobin degradation products [2,6,7,27]. Free haem, iron, reactive oxygen species, and other damage-associated molecular patterns can activate innate immune pathways without active infection [28,29]. Repeated retention may therefore promote oxidative stress, epithelial injury, inflammatory activation, and endometriosis formation within the pouch, potentially contributing to symptoms and impaired reproductive function even when CD138-positive plasma cells are scarce [7,30,31,32].

CD138 identifies plasma-cell infiltration rather than innate sterile inflammation [13,14,15,16,33]; negative or low-level staining therefore does not exclude injury-driven inflammatory activity. Sonography quantifies anatomical dimensions but not blood retention, epithelial disruption, or local inflammatory mediators [3,6,7,27]. Because these mediators were not measured, our data do not demonstrate sterile inflammation but support it as a mechanistically plausible hypothesis that is being investigated prospectively.

CD138 positivity in our cohort was lower than the pooled estimate of approximately 40% in a recent meta-analysis of chronic endometritis in women with isthmocele [12]. Comparisons are limited by differing thresholds, counting methods, patient selection, controls, and specimen origin. Wei et al. reported chronic endometritis in 28.8% of women with CSD versus 19.6% of matched controls [17], and Zhang et al. reported an association with prolonged menstruation [11]. In contrast, Wang et al. found increased infiltration at the scar surface but not in cavity specimens [10], while Gao et al. found no independent association between previous caesarean delivery and chronic endometritis [18].

These conflicting findings may partly reflect different anatomical compartments. Histological studies of CSD have demonstrated epithelial deficiency, fibrosis, vascular abnormalities, adenomyosis, retained blood products, and inflammatory infiltrates directly within the scar [8,9,10,11], whereas the remaining endometrial cavity appears less affected. A cavity biopsy may therefore miss inflammatory activity where blood retention and repetitive tissue injury are most pronounced.

Neither our findings nor the proposed sterile-inflammation model excludes a microbial contribution. We did not perform cultures, molecular pathogen detection, or microbiome analyses. Bacterial colonisation of CSD and altered uterine microbial profiles have been reported [20,21], and a prospective study described microbiome changes after hysteroscopic treatment, although concurrent antibiotics and the lack of a control group limit causal interpretation [22]. Sterile and microorganism-associated mechanisms may therefore coexist.

CD138 positivity did not differ significantly between the two surgical groups despite their different defect dimensions. Patients undergoing niche excision had substantially thinner residual myometrium and deeper defects than those treated hysteroscopically, consistent with established selection principles for CSD surgery [23,24,34]. Thus, anatomical subgroups leading to different surgical approaches had similar rates of endometrial plasma-cell infiltration.

The lack of association between CD138 status and clinical symptoms is also relevant. In contrast to studies linking scar inflammation to prolonged menstruation [10,11], our findings did not identify a distinct symptom pattern associated with endometrial plasma-cell infiltration. Differences in biopsy location and inflammatory markers may contribute to these discrepancies. CSDi symptoms probably arise from overlapping mechanical, vascular, inflammatory, and endometriosis-associated mechanisms [6,7,30].

Endometrial CD138 testing is frequently considered in recurrent pregnancy loss (RPL) and recurrent implantation failure (RIF), where chronic endometritis prevalence varies with diagnostic criteria [35]. Its relevance remains contested: some cohorts found no association with failed transfers or miscarriages [36,37], whereas others reported improved outcomes after antibiotics, particularly in RIF [38,39]. ESHRE does not recommend routine chronic endometritis testing in unexplained RPL [26]. In our cohort, neither RPL nor repeated embryo transfer was associated with CD138 positivity; because few affected patients were CD138-positive, these findings are descriptive. They are nevertheless consistent, with reproductive failure in CSDi being more closely related to the niche than to a CD138-associated endometrial process.

The diagnostic threshold is central to interpretation. Most positive findings represented low-level plasma-cell infiltration, whereas only a small proportion of patients had ≥5 cells/HPF. Low-level plasma-cell infiltration may not predict implantation or live birth, while adverse reproductive outcomes appear more consistently associated with higher counts [13,14,15,16,33]. Reporting the cell-count distribution therefore provides more information than a binary diagnosis and cautions against equating low-level positivity with clinically relevant chronic endometritis. Niche morphology or symptoms alone would not justify routine CD138 testing or empirical antibiotics; decisions should consider standardised pathology, clinical and reproductive context, and, where appropriate, microbiological findings.

The association between CSDi and infertility remains clinically important. Niche-related mechanical and inflammatory mechanisms may impair sperm transport, implantation, and reproductive outcomes [7,31], and meta-analytic evidence indicates that isthmocele is associated with reduced in vitro fertilisation success [32]. Our findings do not exclude an inflammatory contribution because the study was not designed to evaluate implantation, pregnancy, or live-birth outcomes and CD138-positive patients were few.

### Strengths and Limitations

Strengths include the large, clinically well-characterised surgical cohort, uniform endometrial-cavity sampling, detailed sonographic niche measurements, and multivariable adjustment for age, body mass index, parity, and defect size. Several limitations should be acknowledged.

The single-centre design and restriction to symptomatic patients undergoing surgery limit generalisability.

Only 26 patients were CD138-positive and only seven had more than four cells, resulting in limited statistical power and wide confidence intervals. The study therefore cannot exclude modest associations, and the absence of statistical significance should not be interpreted as evidence of equivalence. CD138 assessment varies with the number of high-power fields, tissue area, menstrual-cycle phase, staining protocol, and observer interpretation, and the absence of an internationally accepted threshold remains an important source of heterogeneity [13,14,15,16].

Patients who had undergone diagnostic CD138 testing elsewhere for reproductive indications and received corresponding presurgical treatment were excluded. Exclusion of this small pretreated subgroup may have introduced selection bias and could have led to underestimation of CD138 positivity in the analysed cohort.

Potential confounders including recent antibiotic exposure, recent genital tract infection, menstrual-cycle timing at biopsy, previous hysteroscopy or endometrial polypectomy, and intrauterine device use were not systematically recorded and could therefore not be included in the analyses. Residual confounding by these factors cannot be excluded. Although sonographic assessment was standardised and performed by a single experienced examiner, inter-observer reproducibility could not be evaluated. Hysteroscopic signs of focal endometritis were likewise not systematically assessed using a predefined protocol.

No non-CSD control group, paired scar biopsy, endometrial culture, microbiome analysis, postoperative CD138 assessment, or measurement of sterile inflammatory mediators was available. The cross-sectional design also precludes conclusions about whether plasma-cell infiltration is a cause, consequence, or incidental correlate of CSDi.

## 5. Conclusions

CD138-positive plasma cells were detected in a minority of endometrial cavity biopsies from women undergoing surgery for symptomatic CSD and were not associated with niche morphology, defect size, surgical approach, or clinical symptoms. Endometrial CD138 staining therefore does not appear to reflect anatomical severity or clinical phenotype. Whether CSD-associated inflammation is injury-driven, locally confined, or microorganism-associated requires direct assessment of inflammatory mediators and microbiological findings. Our findings do not support selecting patients for endometrial CD138 testing on the basis of niche subtype or defect size. However, they do not exclude either sterile or microorganism-associated inflammatory mechanisms. Because no endometrial cultures or microbiome analyses were performed, no inference regarding the presence or absence of infection can be drawn.

## Figures and Tables

**Table 1:** Characteristics of the study population (n = 196) by surgical technique. Data are presented as mean ± SD/median (IQR).

Characteristic	n	Total (n = 196)	Niche Excision (n = 147)	Hysteroscopy Only (n = 49)	*p*
Age at surgery, years	196	35.5 ± 4.9/35.9 (32.8–38.6)	35.5 ± 4.2/35.7 (32.7–38.5)	35.4 ± 6.4/36.0 (33.6–38.6)	0.50
Body mass index, kg/m^2^	195	25.9 ± 5.2/24.9 (22.3–28.3)	26.1 ± 5.0/25.2 (22.6–28.6)	25.1 ± 5.6/24.2 (21.5–26.3)	0.054
Residual myometrium before surgery, mm	196	1.58 ± 2.23/0.30 (0.00–2.52)	0.60 ± 0.97/0.00 (0.00–1.10)	4.52 ± 2.36/3.60 (2.70–5.90)	<0.001
Niche depth before surgery, mm	192	6.8 ± 3.8/6.9 (4.7–9.2)	7.3 ± 4.1/7.8 (5.9–9.8)	5.3 ± 2.2/5.4 (3.6–6.6)	<0.001
Proportion of niche in myometrium	171	0.81 ± 0.23/0.87 (0.68–1.00)	0.91 ± 0.14/1.00 (0.85–1.00)	0.54 ± 0.19/0.58 (0.42–0.70)	<0.001
Interval from most recent documented caesarean delivery to surgery, years	176	4.4 ± 2.5/4.0 (3.0–6.0)	4.4 ± 2.6/4.0 (3.0–6.0)	4.4 ± 2.4/4.0 (2.8–6.0)	0.89

**Table 4:** Multivariable logistic regression analyses of factors associated with endometrial CD138 positivity.

Predictor	Model 1 (Niche Depth) OR (95% CI); *p* n = 191	Model 2 (RMT) OR (95% CI); *p* n = 195
Niche depth (mm)	1.00 (0.90–1.13); >0.9	–
Residual myometrium (mm)	–	0.98 (0.70–1.31); 0.9
Surgical technique (Excision via Regensburg Approach vs. HSK)	1.53 (0.55–4.98); 0.4	1.37 (0.30–7.38); 0.7
Age (years)	1.00 (0.92–1.11); >0.9	1.00 (0.92–1.11); >0.9
BMI (kg/m^2^)	0.95 (0.85–1.04); 0.3	0.95 (0.86–1.04); 0.3
Parity	1.34 (0.88–1.96); 0.13	1.35 (0.89–1.97); 0.13

## Data Availability

The data that support the findings of this study are available from the corresponding author upon reasonable request.

## Supplementary Materials

The following supporting information can be downloaded at https://www.mdpi.com/article/10.3390/diagnostics16213472/s1, Supplementary Table S1: Sensitivity analysis using the stricter CD138 threshold (≥5 cells/HPF vs. <5 cells/HPF).

## References

[1] Betran A.P., Ye J., Moller A.B., Souza J.P., Zhang J. (2021). Trends and projections of caesarean section rates: Global and regional estimates. BMJ Glob. Health.

[2] van der Voet L.F., Bij de Vaate A.M., Veersema S., Brölmann H.A., Huirne J.A. (2014). Long-term complications of caesarean section. The niche in the scar: A prospective cohort study on niche prevalence and its relation to abnormal uterine bleeding. BJOG.

[3] Jordans I.P.M., de Leeuw R.A., Stegwee S.I., Amso N.N., Barri-Soldevila P.N., van den Bosch T., Bourne T., Brölmann H.A.M., Donnez O., Dueholm M. (2019). Sonographic examination of uterine niche in non-pregnant women: A modified Delphi procedure. Ultrasound Obstet. Gynecol..

[4] Klein Meuleman S.J., Murji A., van den Bosch T., Donnez O., Grimbizis G., Saridogan E., Chantraine F., Bourne T., Timmerman D., Huirne J.A. (2023). Definition and criteria for diagnosing cesarean scar disorder. JAMA Netw. Open.

[5] Klein Meuleman S.J.M., Verberkt C., Barri P.N., Murji A., Donnez O., Grimbizis G., Saridogan E., Bourne T., Zhang J., Pomorski M. (2025). Prevalence of cesarean scar disorder in patients 3 years after a first cesarean section. Acta Obstet. Gynecol. Scand..

[6] Tsuji S., Nobuta Y., Hanada T., Takebayashi A., Inatomi A., Takahashi A., Amano T., Murakami T. (2023). Prevalence, definition, and etiology of cesarean scar defect and treatment of cesarean scar disorder: A narrative review. Reprod. Med. Biol..

[7] Vissers J., Hehenkamp W., Lambalk C.B., Huirne J.A. (2020). Post-Caesarean section niche-related impaired fertility: Hypothetical mechanisms. Hum. Reprod..

[8] Morris H. (1995). Surgical pathology of the lower uterine segment caesarean section scar: Is the scar a source of clinical symptoms?. Int. J. Gynecol. Pathol..

[9] Higuchi A., Tsuji S., Nobuta Y., Nakamura A., Katsura D., Amano T., Kimura F., Tanimura S., Murakami T. (2022). Histopathological evaluation of cesarean scar defect in women with cesarean scar syndrome. Reprod. Med. Biol..

[10] Wang Y., Han Y., Guo X., Wei Q., Xia Y., Gao L., Wang H., Lu X., Shu J. (2025). Caesarean scar endometrial defects contribute to post-caesarean abnormal uterine bleeding and chronic endometritis: A retrospective case-control study. BJOG.

[11] Zhang J., Huang J., Xu Z., Yang Q., Zeng L., Zhou L., Deng K. (2024). The correlation between chronic endometritis and Caesarean scar diverticulum. J. Reprod. Immunol..

[12] Vidal A., Pape J., Vinayahalingam V., Gulz M., Karrer T., von Wolff M. (2025). High risk of chronic endometritis in isthmocele: A systematic review and meta-analysis. J. Clin. Med..

[13] Kasius J.C., Broekmans F.J.M., Sie-Go D.M.D.S., Bourgain C., Eijkemans M.J.C., Fauser B.C., Devroey P., Fatemi H.M. (2012). The reliability of the histological diagnosis of endometritis in asymptomatic IVF cases: A multicenter observer study. Hum. Reprod..

[14] Kuroda K., Yamanaka A., Takamizawa S., Nakao K., Kuribayashi Y., Nakagawa K., Nojiri S., Nishi H., Sugiyama R. (2022). Prevalence of and risk factors for chronic endometritis in patients with intrauterine disorders after hysteroscopic surgery. Fertil. Steril..

[15] Li Y., Xu S., Yu S., Huang C., Lin S., Chen W., Mo M., Lian R., Diao L., Ding L. (2021). Diagnosis of chronic endometritis: How many CD138-positive cells/high-power field in endometrial stroma affect pregnancy outcome of infertile women?. Am. J. Reprod. Immunol..

[16] Santoro A., Travaglino A., Inzani F., Angelico G., Raffone A., Maruotti G.M., Straccia P., Arciuolo D., Castri F., D’alessandris N. (2023). The role of plasma cells as a marker of chronic endometritis: A systematic review and meta-analysis. Biomedicines.

[17] Wei L., Xu C., Zhao Y., Zhang C. (2023). Higher prevalence of chronic endometritis in women with cesarean scar defect: A retrospective study using propensity score matching. J. Pers. Med..

[18] Gao Y., Xu G., Yang M., Chen K., Wang Y. (2024). Risk factors of chronic endometritis in women who have undergone hysteroscopy: A prospective nested case-control study. Sci. Rep..

[19] Born T., Krejci K., Rauh M., Cole G., Kappelmeyer M., Vural M., Köninger A. (2025). Extragenital endometriosis associated with uterine scar defects. Eur. J. Obstet. Gynecol. Reprod. Biol. X.

[20] Yang X., Pan X., Cai M., Zhang B., Liang X., Liu G. (2021). Microbial flora changes in cesarean section uterus and its possible correlation with inflammation. Front. Med..

[21] Hsu I., Hsu L., Dorjee S., Hsu C.C. (2022). Bacterial colonization at caesarean section defects in women of secondary infertility: An observational study. BMC Pregnancy Childbirth.

[22] Sezer E., Cevrioglu A.S., Ozozen E., Koroglu M., Uslu Yuvaci H., Aslan M.M., Bostanci M.S., Akdemir N. (2026). Association between hysteroscopic treatment of cesarean scar disorder and changes in the endometrial microbiome and clinical outcomes: A prospective observational study. Reprod. Med. Biol..

[23] Vitale S.G., Ludwin A., Vilos G.A., Torok P., Tesarik J., Vitagliano A., Lasmar R.B., Chiofalo B. (2020). From hysteroscopy to laparoendoscopic surgery: What is the best surgical approach for symptomatic isthmocele? A systematic review and meta-analysis. Arch. Gynecol. Obstet..

[24] Mashiach R., Burke Y.Z. (2021). Optimal isthmocele management: Hysteroscopic, laparoscopic, or combination. J. Minim. Invasive Gynecol..

[25] Vervoort A.J.M.W., Uittenbogaard L.B., Hehenkamp W.J.K., Brölmann H.A.M., Mol B.W.J., Huirne J.A.F. (2015). Why do niches develop in Caesarean uterine scars? Hypotheses on the aetiology of niche development. Hum. Reprod..

[26] Bender Atik R., Christiansen O.B., Elson J., Kolte A.M., Lewis S., Middeldorp S., Mcheik S., Peramo B., Quenby S., ESHRE Guideline Group on RPL (2023). ESHRE guideline: Recurrent pregnancy loss: An update in 2022. Hum. Reprod. Open.

[27] Dominguez J.A., Pacheco L.A., Moratalla E., Carugno J.A., Carrera M., Perez-Milan F., Caballero M., Alcázar J.L. (2023). Diagnosis and management of isthmocele (cesarean scar defect): A SWOT analysis. Ultrasound Obstet. Gynecol..

[28] Chen G.Y., Nuñez G. (2010). Sterile inflammation: Sensing and reacting to damage. Nat. Rev. Immunol..

[29] Dutra F.F., Bozza M.T. (2014). Heme on innate immunity and inflammation. Front. Pharmacol..

[30] Mohr-Sasson A., Dadon T., Brandt A., Shats M., Axcelrod M., Meyer R., Zajicek M., Haas J., Mashiach R. (2023). The association between uterine scar defect (niche) and the presence of symptoms. Reprod. BioMed Online.

[31] Nobuta Y., Tsuji S., Kitazawa J., Hanada T., Nakamura A., Zen R., Amano T., Murakami T. (2022). Decreased fertility in women with cesarean scar syndrome is associated with chronic inflammation in the uterine cavity. Tohoku J. Exp. Med..

[32] Vitagliano A., Cicinelli E., Viganò P., Sorgente G., Nicolì P., Busnelli A., Dellino M., Damiani G.R., Gerli S., Favilli A. (2024). Isthmocele, not cesarean section per se, reduces in vitro fertilization success: A systematic review and meta-analysis of over 10,000 embryo transfer cycles. Fertil. Steril..

[33] Herlihy N.S., Klimczak A.M., Titus S., Scott C., Hanson B.M., Kim J.K., Seli E., Scott R.T. (2022). The role of endometrial staining for CD138 as a marker of chronic endometritis in predicting live birth. J. Assist. Reprod. Genet..

[34] Vervoort A.J.M.W., Vissers J., Hehenkamp W.J.K., Brölmann H.A.M., Huirne J.A.F. (2018). The effect of laparoscopic resection of large niches in the uterine caesarean scar on symptoms, ultrasound findings and quality of life: A prospective cohort study. BJOG.

[35] Hue H.J., Choi H., Lee H.K., Lee J.R., Jee B.C., Choo C.W., Kim S.K. (2024). Prevalence and confounders of chronic endometritis diagnosed using CD138 in patients with recurrent implantation failure. Clin. Exp. Reprod. Med..

[36] Zargar M., Ghafourian M., Nikbakht R., Mir Hosseini V., Moradi Choghakabodi P. (2020). Evaluating chronic endometritis in women with recurrent implantation failure and recurrent pregnancy loss by hysteroscopy and immunohistochemistry. J. Minim. Invasive Gynecol..

[37] Xu Y., Kwan P.Y., Chen W., Zhang Q., Zhou C., Mol B.W., Xu Y., Gu F. (2025). The effect of antibiotic treatment on pregnancy outcomes in patients with mild chronic endometritis undergoing in vitro fertilization. Fertil. Steril..

[38] Xie Q., Zhao C., Jiang W., Li X., Ni D., Chen Y., Li X., Hua X., Shen R., Ling X. (2024). Antibiotics improve reproductive outcomes after frozen-thaw embryo transfer for chronic endometritis treatment, especially in those with repeated implantation failure. BMC Womens Health.

[39] Xiong Y., Chen Q., Chen C., Tan J., Wang Z., Gu F., Xu Y. (2021). Impact of oral antibiotic treatment for chronic endometritis on pregnancy outcomes in the following frozen-thawed embryo transfer cycles of infertile women: A cohort study of 640 embryo transfer cycles. Fertil. Steril..

