# Supplementary material for: CD138-Positive Plasma Cells in the Endometrium of Women with a Caesarean Scar Defect
Source: Diagnostics (Basel). 2026 Oct 24;16(21):3472. doi: 10.3390/diagnostics16213472 (PMC13761813; doi:10.3390/diagnostics16213472)
Supplement: Supplementary file 1 [file diagnostics-16-03472-s001.zip › diagnostics-4613630-supplementary.pdf]

Supplementary Table S1

Sensitivity analysis using the stricter CD138 threshold (≥5 cells/HPF vs. <5 cells/HPF)

Sensitivity analyses were restricted to univariable comparisons because only seven patients met the ≥5 cells/HPF threshold. Continuous variables are presented as median (IQR); effect estimates are differences in medians (≥5 minus <5) with bootstrap 95% confidence intervals. Categorical variables are presented as n/N (%); effect estimates are odds ratios (ORs) with exact 95% confidence intervals. P values are from Mann–Whitney U tests or Fisher's exact tests, as appropriate. For uterine position, an overall exact test across categories was used. Analyses were exploratory and no multiplicity adjustment was applied.

**Variable**	**≥5 cells/HPF**	**<5 cells/HPF**	**Effect estimate (95% CI)**	**p**
RMT, mm	1.00 (0.00–3.85)<br>(n=7)	0.00 (0.00–2.50)<br>(n=189)	Median difference 1.00 (-1.00 to 5.50)	0.617
Niche depth, mm	6.50 (5.20–7.60)<br>(n=7)	7.00 (4.70–9.30)<br>(n=185)	Median difference -0.50 (-2.50 to 1.70)	0.792
Proportion of niche in myometrium	0.89 (0.49–1.00)<br>(n=7)	0.87 (0.69–1.00)<br>(n=164)	Median difference 0.02 (-0.49 to 0.17)	0.722
Uterine position, overall	Anteflexion 4/7 (57.1%)<br>Retroflexion 3/7 (42.9%)<br>Other 0/7 (0%)	Anteflexion 101/188 (53.7%)<br>Retroflexion 60/188 (31.9%)<br>Other 27/188 (14.4%)	—	0.755
Inclusion cyst	0/7 (0%)	43/176 (24.4%)	OR 0.00 (0.00–2.25)	0.202
Concomitant endometriosis	4/7 (57.1%)	105/180 (58.3%)	OR 0.95 (0.16–6.69)	1.000
Niche excision	5/7 (71.4%)	142/189 (75.1%)	OR 0.83 (0.13–8.97)	1.000
Hypermenorrhea	2/7 (28.6%)	26/182 (14.3%)	OR 2.39 (0.22–15.54)	0.277
Abnormal discharge	2/6 (33.3%)	59/156 (37.8%)	OR 0.82 (0.07–5.95)	1.000
Infertility	5/7 (71.4%)	134/189 (70.9%)	OR 1.03 (0.16–11.08)	1.000
Dysmenorrhea	2/7 (28.6%)	95/188 (50.5%)	OR 0.39 (0.04–2.48)	0.445
Dyspareunia	1/7 (14.3%)	72/187 (38.5%)	OR 0.27 (0.01–2.28)	0.258
Postmenstrual spotting	6/7 (85.7%)	138/188 (73.4%)	OR 2.17 (0.25–101.97)	0.679
RPL-related history	1/5 (20.0%)	18/135 (13.3%)	OR 1.62 (0.03–17.62)	0.523
≥2 previous embryo transfers	2/5 (40.0%)	36/82 (43.9%)	OR 0.85 (0.07–7.87)	1.000

Abbreviations: CI, confidence interval; HPF, high-power field; OR, odds ratio; RMT, residual myometrial thickness; RPL, recurrent pregnancy loss-related history. “Other” uterine positions comprise straight and torqued
